# De novo assembly of transcriptome and genome-wide identification reveal GA_3_ stress-responsive WRKY transcription factors involved in fiber formation in jute (*Corchorus capsularis*)

**DOI:** 10.1186/s12870-020-02617-8

**Published:** 2020-09-01

**Authors:** Lilan Zhang, Xuebei Wan, Yi Xu, Sylvain Niyitanga, Jianmin Qi, Liwu Zhang

**Affiliations:** 1 Key Laboratory of Ministry of Education for Genetics, Breeding and Multiple Utilization of Crops / Fujian Key Laboratory for Crop Breeding by Design / College of Agriculture, Fujian Agriculture and Forestry University, Fuzhou, 350002 China; 2 Experiment Station of Ministry of Agriculture and Rural Affairs for Jute and Kenaf in Southeast China / Fujian Public Platform for Germplasm Resources of Bast Fiber Crops / Fujian International Science and Technology Cooperation Base for Genetics, Breeding and Multiple Utilization Development of Southern Economic Crops, Fujian Agriculture and Forestry University, Fuzhou, 350002 Fujian China; 3 Center for Genomics and Biotechnology, Haixia Institute of Science and Technology, Fujian Agriculture and Forestry University, Fuzhou, 350002 China

**Keywords:** Jute, WRKY transcription factor, GA_3_ stress, Expression pattern, Phylogenetic analysis

## Abstract

**Background:**

WRKY is a group of transcription factors (TFs) that play a vital role in plant growth, development, and stress tolerance. To date, none of jute WRKY (CcWRKY) genes have been identified, even if jute (*Corchorus capsularis*) is one of the most important natural fiber crops in the world. Little information about the WRKY genes in jute is far from sufficient to understand the molecular mechanism of bast fiber biosynthesis.

**Results:**

A total of 244,489,479 clean reads were generated using Illumina paired-end sequencing. De novo assembly yielded 90,982 unigenes with an average length of 714 bp. By sequence similarity searching for known proteins, 48,896 (53.74%) unigenes were annotated. To mine the CcWRKY TFs and identify their potential function, the search for *CcWRKYs* against the transcriptome data of jute was performed, and a total of 43 *CcWRKYs* were identified in this study. The gene structure, phylogeny, conserved domain and three-dimensional structure of protein were analyzed by bioinformatics tools of GSDS2.0, MEGA7.0, DNAMAN5.0, WebLogo 3 and SWISS-MODEL respectively. Phylogenetic analysis showed that 43 *CcWRKYs* were divided into three groups: I, II and III, containing 9, 28, and 6 members respectively, according to the WRKY conserved domain features and the evolution analysis with *Arabidopsis thaliana*. Gene structure analysis indicated that the number of exons of these *CcWRKYs* varied from 3 to 11. Among the 43 *CcWRKYs*, 10, 2, 2, and 14 genes showed higher expression in leaves, stem sticks, stem barks, and roots at the vigorous vegetative growth stage, respectively. Moreover, the expression of 21 of 43 *CcWRKYs* was regulated significantly with secondary cell wall biosynthesis genes using FPKM and RT-qPCR by GA_3_ stress to a typical GA_3_ sensitive dwarf germplasm in comparison to an elite cultivar in jute. The *Cis*-element analysis showed that promoters of these 21 *CcWRKYs* had 1 to 4 cis-elements involved in gibberellin-responsiveness, suggesting that they might regulate the development of bast fiber in response to GA_3_ stress.

**Conclusions:**

A total of 43 *CcWRKYs* were identified in jute for the first time. Analysis of phylogenetic relationship and gene structure revealed that these *CcWRKYs* might have a functional diversity. Expression analysis showed 21 TFs as GA_3_ stress responsive genes. The identification of these *CcWRKYs* and the characterization of their expression pattern will provide a basis for future clarification of their functions in bast fiber development in jute.

## Background

The WRKY gene family are transcription factors that exists only in plants. It is mainly involved in transcriptional regulation and signal transduction processes in plants [1]. In the former one, WRKY transcription factors bind specific DNA sequences to repress or activate transcription of numerous important genes [2, 3]. The conserved WRKY domain comprises 60 aa residues and a conserved WRKYGQK hexapeptide sequence within the WRKY domain is normally accompanied by a C2HC-type or C2H2 zinc-finger structure. According to the number of WRKY domains and the type of zinc finger structure, the WRKY family can be divided into 3 groups: Group I, Group II and Group III [4]. Group II could be further divided into five subgroups: II-a, II-b, II-c, II-d and II-e. Group I contains two WRKY domains and C_2_H_2_ zinc finger structure, Group II contains a WRKY domain and a C_2_H_2_ zinc finger structure, and Group III contains a WRKY domain and a C_2_-H-C zinc finger structure [5].

Since the first WRKY gene (*SPF1*) was cloned from sweet potato [6], WRKY genes have been found in many plants, such as *Arabidopsis* [7], rice [8], barley [9], rapeseed [10], soybean [11], corn [12], potato [13], tobacco [14], poplar [15], cotton [16], cucumber [5] and grape [17]. At present, WRKY transcription factors have been found to play an important role in plant defense response to environmental stresses and growth development. The WRKY70 TF (transcription factor), is an essential component in JA (jasmonic) and SA (salicylic acid)-mediated signaling pathways [18]. Salicylic acid activates the WRKY70, while jasmonic acid represses its expression [18]. In *Arabidopsis*, some WRKY transcription factors are positive regulators of ABA (Abscisic acid)-mediated stomatal closure and hence drought responses [19]. *AtWRKY75* in *Arabidopsis thaliana* and *OsWRKY31* in rice are both related to the growth of lateral root [20, 21]. In aleurone cells, ABA-induced WRKY genes synergistically interact in controlling GAMYB-mediated abscisic acid and gibberellin signaling and thus establishing a novel process for abscisic acid and gibberellin signaling cross-talk [22].

Jute (*Corchorus capsularis* L) is a diploid (2*n* = 14) natural fiber plant, which belongs to the Malvaceae family [23]. It is mainly cultivated in Bangladesh, India, and China. The bast fiber of jute is mainly used to make fabrics, ropes and threads, which is an indispensable part in human’s life. Due to the reason that plant fibers are renewable, eco-friendly, and 100% biodegradable, more and more people are interested in plant fiber textiles and production technology [24]. Jute gained considerable attention in recent decades.

It has long been known that WRKY genes play an important role in plant growth, development, stress tolerance, especially in fiber development [25]. And bioactive gibberellins (GAs) are also a type of important plant growth regulators, which play the key roles in stem development. However, none of WRKY (CcWRKY) genes has been identified in jute so far. Therefore, it is imperative to identify and analyze the WRKY transcription factors in jute after the release of the draft genome and transcriptome [23]. The aims of this study were to identify the *CcWRKYs* and analyze their expression pattern in different tissues, especially bast fiber in response to GA_3_ stress.

## Methods

### Plant materials and treatments

Two genotypes, a typical GA_3_ sensitive dwarf germplasm (“Aidianyehuangma”) and an elite cultivar (“Huangma 179”), were used as plant materials in this study. The two genotypes of jute were grown in the farm of Fujian Agriculture and Forestry University on May 1st, 2017. When 10 days after sowing (DAS), hypocotyls were independently sampled from three plants of “Huangma 179”. The leaves, roots, stem barks and stem sticks were independently collected from three plants of “Huangma 179” when 60 DAS, a vegetative growth period for jute. And stem barks were independently sampled from three plants of “Aidianyehuangma”. When 120 DAS, the stem barks were independently collected from three plants of “Huangma 179”.

To explore the effects of GA_3_ stress on bast fiber in jute, “Huangma 179” was used as the control variety and “Aidianyehuangma” as the test variety. According to the method of MS medium [26], the standard medium (CK) and 0.1 mg·L^− 1^ GA_3_ (GA3) exogenous hormone medium were prepared. An elite cultivar “Huangma 179” and a typical GA_3_ sensitive dwarf germplasm “Aidianyehuangma” were used in this study. Thirty seeds were planted under MS medium in each bottle, and the length of hypocotyl was measured when the first leaf was grown.

To identify the expression of *CcWRKYs* in bast fiber in response to GA_3_ stress, six plants of the two genotypes were treated with 100 mg·L^− 1^ exogenous GA_3_ on 60 DAS. After 4 h and 72 h of the GA_3_ stress treatment, the stem barks of each plant were obtained from “Huangma 179” and “Aidianyehuangma” respectively. Also, three plants of the two genotypes were sampled separately as control. Three samples from each tissue were immediately frozen in liquid nitrogen and stored at − 80 °C separately as three biological replicates for the following RNA isolation (Additional file 1: Table S1).

### Transcriptome sequencing assembly

Total RNA was isolated from three separate collected samples using Plant Total RNA Isolation kit (Tiandz Inc.; Beijing, China), respectively. RNAs of each sample tissue were equally mixed together for libraries construction as well as for Illumina sequencing which was performed by a company, Beijing Novogene Biological Information Technology Co., Ltd., Beijing, China (https:// www.novogene.cn/)^.^ In short, 10 μg of good quality RNA was collected for transcriptome library construction. mRNAs of 12 sequencing libraries (Additional file 1: Table S1) was purified from total RNA of each sample tissues using Poly-T oligo-attached magnetic beads. These mRNAs were then fragmented with divalent cations under raised temperature in Illumina patented fragmentation buffer. The superscript II and random oligonucleotides were utilized to synthesize first cDNA strand while RNase H and Polymerase I were used for getting the second cDNA strand. Fragments holding adaptors were selected with gel purification as well as Illumina polymerase chain reaction (PCR) amplification to produce cDNA library. Systems like Agilent Bioanalyzer 2100 and AMPure XP system were applied to quantify and purify the PCR products respectively. Index-coded clustering of samples was conducted using the cBot Cluster Generation System with Illumina (TruSeq PE Cluster Kit v3-cBot-HS) referring to the manufacturer’s recommendations. After cluster production, transcriptome sequencing was carried out using Hiseq 2000 platform Illumina with 90 bp paired-ends reads.

The raw data (sequence) reads were filtered for quality by trimming reads holding adapter (poly-N and low-quality sequence reads) in Perl script house. The assembled sequence data with high quality were de novo assembled into a transcriptome using Trinity (a short read- assembly) program containing all default settings for all considerations and mapped to transcripts. Transcripts that had less than 10× coverage were removed and finally good quality raw data were deposited in the NCBI Sequence Read Archive (SRA) database (BioProject ID: PRJNA555734) with accession codes of SAMN12327429 to SAMN12327440.

Gene function was annotated by homology search using BLASTx with an E value < 10^− 5^) against diverse databases in NCBI including Pfam, annotated protein families (http://pfam.janelia.org/); Swiss-Prot (http://www.expasy.ch/sprot); nr, non-redundant protein sequences (http://www.ncbi.nlm.nih.gov); GO, gene ontology (http://www.geneontology.org). GO and nr annotations carried out with Blast2GO program. Gene Ontology functional categorizations were carried out by the WEGO program. Unigene sequences were also aligned to Clusters of Orthologous Groups of Proteins (COG/KOG, (http://www.ncbi.nlm.nih.gov/COG) to predict and classify possible functions. Pathway assignments were performed as per KEGG, Kyoto Encyclopedia of Genes and Genomes pathway database (http://www.genome.jp/kegg).

### Identification, sequence alignment and gene structure analysis of *CcWRKYs*

The assembly of CcWRKY genes and ORF analysis were performed according to Islam et al. [23] and Zhang et al. [27]. Full-length amino acids sequences of all WRKY proteins in *Arabidopsis thaliana* (http://www.arabidopsis.org/) were used as query sequences. A BLASTP search was performed with an *E*-value threshold of 10^− 6^. All the potential CcWRKY genes identified from BLAST searches were only accepted if they contained the WRKY domain. And the conserved domains of predicted WRKY were confirmed using multiple sequence alignments. Further, conservative domain prediction using Pfam was conducted to ensure that all candidate genes contain WRKY conserved domains [28]. Candidate WRKYs were also checked by searching for WRKY domains in the candidate amino acids sequences using SWISS-MODELL (http://swissmodel.expasy.org/) [29]. The intron/exons information of CcWRKYs were obtained by loading both the full-length protein sequences and their related coding sequences into GSDS (Gene Structure Display Server) version 2.0. Base on homologous proteins in *Arabidopsis* as query sequences, secondary cell wall biosynthesis genes in jute were also identified by local BLASTP.

### Phylogenetic analysis of *CcWRKYs*

Due to the variable lengths of the complete protein sequences of the WRKY genes, WRKY domains extracted from the predicted proteins were used to draw the phylogenetic tree. A neighbor joining evolutionary tree was constructed by MEGA7 software [30]. The p-distance method was used to compute the evolutionary distances, which were used to estimate the number of amino acid substitutions per site. Conducting 1000 bootstrap sampling steps [12] was used to establish the reliability of each tree. The phylogenetic tree was divided into the different groups on the basis of *Arabidopsis* annotations and classification. WRKY family sequences from *Arabidopsis* were downloaded from TAIR (http://www.arabidopsis.org/).

### RNA extraction and gene expression analysis

Total RNA was isolated from three separate collected samples using Plant Total RNA Isolation kit (Tiandz Inc.; Beijing, China), respectively. The cDNAs were transcribed from RNAs using the GoScript™ reverse transcription system (Promega, Madison, USA) referring to manufacturer’s directions. The *Cc actin* gene was used as a reference gene to normalize the expression of CcWRKY [31]. Three independent reactions were performed for putative CcWRKY genes using the Fast Start Universal SYBR® Green Master (ROX) with a 7500 real-time PCR machine (ABI) referring to manufacturer’s directions. The reaction systems were: 0.4 μl of right primer (10 μM), 0.4 μl of left primer (10 μM), 10 μl of GoTaq® qPCR Master Mix, 7.2 μl of Nuclease-Free Water, and 2 μl of cDNA. Amplification conditions were programmed as follows: 30 s of denaturation at 95 °C, followed by 15 s of 40 cycles at 95 °C, and 30 s at 60 °C, with a melting curve temperature ranged from 55 to 99 °C. Each reaction was carried out in three biological replicates and the relative gene expression was determined with 2^–*△△CT*^ method [32]. Three biological repeats with two technical replicates of each sample were performed to acquire reliable results. Data are the mean ± standard deviation of three independent experiments. Error bars indicate standard deviation of the means.

Gene expression levels were calculated as FPKM according to the length of the gene and reads count mapped to this gene: FPKM = total exon fragments/ [mapped reads (millions) × exon length (kb)]. The expression analysis of *CcWRKYs* was performed according to the FRKM value of different genes in different tissues, i.e., Hypocotyl-10d, Leaf-60d, Root-60d, Stem bark-60d, Stem stick-60d, and Stem bark-120d (Additional file 1: Table S1). After GA_3_ stress, an elite cultivar “Huangma 179” was used as a reference variety to estimate the expression of *CcWRKYs* and related genes while a typical GA_3_ sensitive dwarf germplasm “Aidianyehuangma” as a test germplasm. The heatmaps were created by R language based on the transformed data of log_2_^(FPKM + 1)^ values. The differentially expressed genes among the treated samples were estimated by referring to the standard of FDR < 0.05 & |log2(fold change) | > 1. All data analyses were conducted using SPSS Statistics 20. The *cis*-elements within the region of 5 kb upstream promoters of *CcWRKYs* were predicted and analyzed by PlantCARE.

## Results

### De novo assembly of transcriptome

**Fig. 1:**
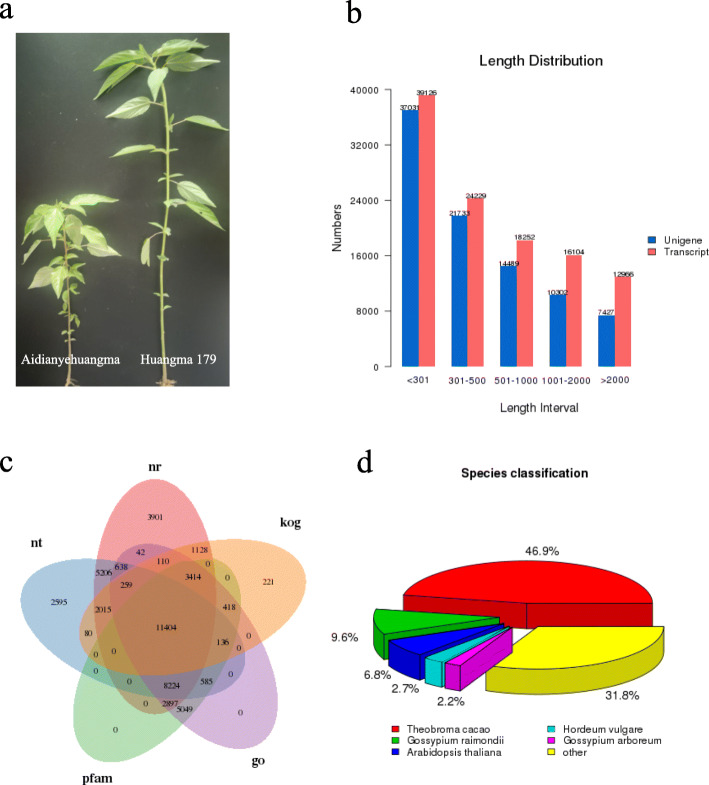
Summary of sequencing data quality. **a** An elite cultivar (“Huangma 179”) and a typical GA_3_ sensitive dwarf germplasm (“Aidianyehuangma”). **b** Distribution of assembled unigene and transcript lengths in jute. The vertical axis indicates the number of unigenes and transcripts, while the horizontal axis indicates the length of unigenes and transcripts. **c** The venn diagram of unigenes annotated in the public databases. KOG: clusters of orthologous groups of proteins, GO: gene ontology, Nr: non-redundant protein sequences, Pfam: annotated protein family, Nt: non-redundant nucleotide sequences. **d.** Percentage numbers of the five most abundant annotated species

To obtain and characterize the expression pattern of GA_3_ stress-responsive WRKY transcription factors in jute, 12 sequencing libraries was from hypocotyls, leaves, roots, stem bark and stem stick at different growth stages between two genotypes, a typical GA_3_ sensitive dwarf germplasm (“Aidianyehuangma”) and an elite cultivar (“Huangma 179”) (Fig. 1a). By Illumina paired-end sequencing, 244,489,479 clean reads comprised of 44.13% GC ratio, and 93.81% Q30 bases were found (Additional file 2: Table S2). Moreover, 110,677 overall transcripts comprised of N50 length of 1710 bp, and mean length of 862 bp, were de novo assembled by trinity program (Fig. 1b**,** Additional file 3: Table S3, Additional file 4: Table S4). Of these transcripts, 73.73% were in the length range of 200 bp to 1000 bp. Further, 90,982 unigenes had N50 length of 1357 bp, and a mean length of 714 bp were assembled from transcripts (Fig. 1b**,** Additional file 3: Table S3, Additional file 4: Table S4). The length of unigenes differed from 201 bp to 15,570 bp. 58,764 unigenes possessed lengths ranged from 201 to 500 bp and 14,489 unigene lengths ranged from 501 to 1000 bp, whereas 17,729 unigenes (19.49%) lengths were greater than 1000 bp (Additional file 3: Table S3).

To evaluate the quality of identified unigenes, de novo assembled trancriptome sequences by trinity were taken as reference sequence. All the obtained clean sequence reads were mapped to the assembled unigenes by RSEM software. Concerning these assembled unigenes functional annotations, the sequence homology search was conducted against Nr (non-redundant protein sequences) at different databases in NCBI using BLASTx with an E-value of 10^− 5^ (Fig. 1c, Additional file 5: Table S5). Among a total of 90,982 unigenes, 39,238 (43.12%) and 33,410 (36.72%) showed significant homology to common genes available in SwissProt and non-redundant protein databases, respectively. Together, 48,896 (53.74%) unigenes unigenes were annotated in not less than one public databases like GO, Nr, KOG, KO, PFAM, or Swiss Prot. Finally, number of sequences from various species matched to that of Jute were determined from the annotation feature (Additional file 6: Table S6). As indicated in Fig. 1d, the three most abundant species were *Theobroma cacao* (11,457, 46.9%), *Hordeum vulgare* (11,071, 9.6%), and *Gossypium raimondii* (8274, 6.8%), representing about 63.3% of the total annotated species.

### Identification of full-length *CcWRKYs* and phylogenetic analysis

To get complete WRKY members in jute (*CcWRKY*), BLASTp search was conducted using *Arabidopsis* full-length WRKY protein sequences from TAIR (http://www.arabidopsis.org) as query. And Pfam was used to detect their conserved domain. A total of 43 candidate genes containing WRKY domain (named as CcWRKY) were identified, which is shown in Table 1.

**Table 1:** Details of the CcWRKY genes, their homologues in other species, the tissues in which they are expressed, and their responses to GA_3_ stress

GeneBank ID	The similar jute Genebank ID		Gene length (bp)	Predicted Protein (aa)	The homologous genes		Major tissue expressed	Stress response (GA_3_)	Subgroup
	GeneBank ID	RS(%)			GeneBank ID	RS(%)			
Ccv40018590	OMO63625.1	96	2351	367	XP_007011366.2(Tc)	78	Leaf-60d	Down	III
Ccv40018580	OMO66745.1	96	2506	315	XP_017985156.1(Tc)	69	Stem bark-60d	Up	III
Ccv40001440	OMO76839.1	98	2757	585	XP_007026134.2(Tc)	82	Stem bark-120d	Up	II-b
Ccv40020010	OMP05343.1	86	5580	304	XP_012453828.1(Gr)	74	Leaf-60d	Up	II-c
Ccv40016590	OMO83201.1	100	2990	495	XP_017978088.1(Tc)	73	Stem stick-60d	Down	II-e
Ccv40017690	OMO93890.1	100	2262	323	XP_017978107.1(Tc)	86	Root-60d	Down	II-c
Ccv40022560	OMO96280.1	97	2915	348	XP_007030538.1(Tc)	85	Root-60d	Down	II-d
Ccv40015220	OMO85692.1	100	2195	321	EOY26052.1(Tc)	91	Stem stick-60d	Up	II-a
Ccv40005710	OMO76157.1	100	1753	288	XP_017978072.1(Tc)	90	Root-60d	Up	II-e
Ccv40044380	OMO49660.1	100	2251	356	XP_007048873.2(Tc)	80	Leaf-60d	Up	II-c
Ccv40032460	OMO55888.1	100	4295	247	XP_007048165.1(Tc)	86	Root-60d	Up	II-c
Ccv40047080	OMO62542.1	94	3097	540	EOX93439.1(Tc)	75	Stem bark-120d	Up	I
Ccv40045520	OMO88967.1	94	3033	688	XP_021274893.1(Hu)	79	Root-60d	Down	II-b
Ccv40037140	OMO56614.1	100	5352	371	XP_007047365.2(Tc)	90	Stem bark-60d	Up	II-d
Ccv40052670	OMO82944.1	100	3505	597	EOY34631.1(Tc)	91	Stem bark-120d	Up	I
Ccv40064170	OMO51304.1	100	1923	415	XP_017972970.1(Tc)	68	Root-60d	Down	II-e
Ccv40065880	OMO49950.1	100	3807	634	EOY21754.1(Tc)	80	Leaf-60d	–	II-b
Ccv40064890	OMO75947.1	100	2128	391	XP_021289025.1(Hu)	72	Leaf-60d	Down	III
Ccv40068880	OMO93415.1	90	6815	358	EOY01748.1(Tc)	75	Stem bark-120d	Up	II-c
Ccv40068550	OMO93362.1	100	3898	531	XP_007045981.2(Tc)	86	Leaf-60d	Down	I
Ccv40090780	OMO61573.1	100	10,945	761	XP_007011727.2(Tc)	80	Stem bark-120d	Up	II-a
Ccv40090200	OMO86139.1	100	2932	341	EOY29233.1(Tc)	94	Root-60d	Up	II-d
Ccv40091890	OMO61754.1	94	3748	515	XP_021277197.1(Hu)	84	Stem bark-120d	Down	I
Ccv40100130	OMO83687.1	99	4632	561	XP_007020620.2(Tc)	91	Root-60d	Down	I
Ccv40098960	OMO83839.1	96	4286	763	XP_021287555.1(Hu)	87	Stem bark-120d	Down	I
Ccv40105280	OMO79927.1	95	2186	541	XP_021286562.1(Hu)	73	Hypocotyl-10d	Down	II-b
Ccv40100860	OMO83490.1	97	3070	604	XP_021287397.1(Hu)	72	Hypocotyl-10d	Up	II-b
Ccv40120290	OMO53862.1	100	2609	252	XP_017975966.1(Hu)	75	Leaf-60d	Up	III
Ccv40139610	OMO72606.1	93	4237	513	XP_021289195.1(Hu)	84	Leaf-60d	Down	I
Ccv40136560	OMO85052.1	100	2681	586	XP_017977471.1(Tc)	83	Stem bark-120d	Up	I
Ccv40154010	OMO79587.1	100	1321	318	XP_007040356.2(Tc)	76	Root-60d	–	II-e
Ccv40154680	OMO79733.1	100	2066	360	XP_021278172.1(Hu)	87	Stem bark-120d	Up	III
Ccv40151700	OMP02240.1	100	1998	294	XP_021284777.1(Hu)	79	Hypocotyl-10d	Down	II-d
Ccv40168680	OMO64133.1	96	3719	534	XP_021281139.1(Hu)	83	Leaf-60d	Down	II-b
Ccv40170690	OMO64390.1	100	1985	344	EOX95862.1(Tc)	90	Root-60d	Up	II-e
Ccv40170160	OMO64319.1	100	1854	337	XP_007051596.2(Tc)	85	Stem bark-120d	Up	III
Ccv40167830	OMO64003.1	100	2345	285	XP_017969931.1(Tc)	85	Root-60d	Down	II-e
Ccv40167950	OMO64022.1	100	1899	322	XP_021279743.1(Hu)	88	Root-60d	Down	II-c
Ccv40173800	OMO78385.1	99	2664	368	XP_007050397.2(Tc)	93	Root-60d	Down	II-d
Ccv40178630	OMO78893.1	100	2433	366	XP_007017155.2(Tc)	91	Stem bark-120d	Up	II-d
Ccv40212000	OMO79225.1	100	2756	581	XP_021297669.1(Hu)	79	Root-60d	Down	II-b
Ccv40227280	OMO53484.1	100	2278	365	EOY06768.1(Tc)	80	Stem bark-120d	Up	II-c
Ccv40228940	OMO79363.1	100	4477	513	XP_021297553.1(Hu)	90	Leaf-60d	Up	I

Note: *Tc Theobroma cacao*, *Hu Herrania umbratica*, *Gr Gossypium raimondii*, *RS* indicated the ratio of similarity of the protein sequence

**Fig. 2:**
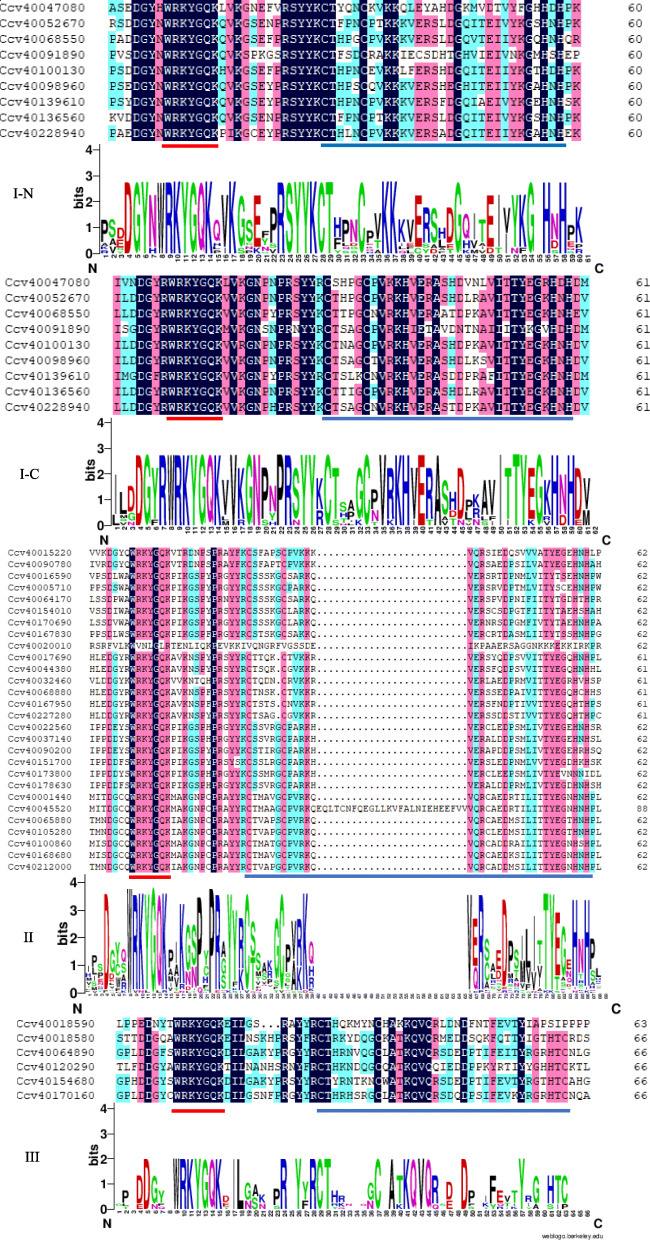
Sequence analysis of the WRKY conserved domain in jute WRKY transcription factors

To further evaluate these *CcWRKYs*, the conservative domains were identified using DNAMAN5.0 software and the conservative structure was performed by Weblogo, as shown in Fig. 2 and Additional file 7. The conserved domains of *CcWRKYs* could be divided into three groups: I, II and III, including 9, 28 and 6 members respectively. Group I, which could be further divided into I-C and I-N subgroups, contains two WRKY domains and zinc finger structures, and the zinc finger structure is CX_4_C_22-23_HXH. Group II could be further divided into subgroups II-a, II-b, II-c, II-d and II-e, with 2, 7, 7, 6 and 6 members, respectively. The heptapeptide domain and zinc finger structure of WRKY at C-terminal in the subgroup of II-a, II-b, II-d and II-e were WRKYGQK and CX_5_C_23_HXH, while the heptapeptide domain and zinc finger structure of WRKY at C-terminal in the subgroup of II-c were WRKYGQK and CX_4_C_23_HXH. Group III contains the heptapeptide domain and the zinc finger structure of WRKY at C-terminal is WRKYGQK and CX_7_C_23_HXC.

Moreover, the present results showed that there are still mutations in its protein sequence, even though WRKY transcription factor has a much conserved WRKY domain. Among the 43 *CcWRKYs* identified in jute, the conserved domain of one gene (WRKYGQK) and the zinc finger structure of four genes were all mutated (Additional file 7: Table S7). These variations indicated that some variations still occur in its WRKY domain despite the structurally high conserved WRKY gene family, which also illustrated that the plant WRKY gene family had a functional diversity in the evolutionary process.

**Fig. 3:**
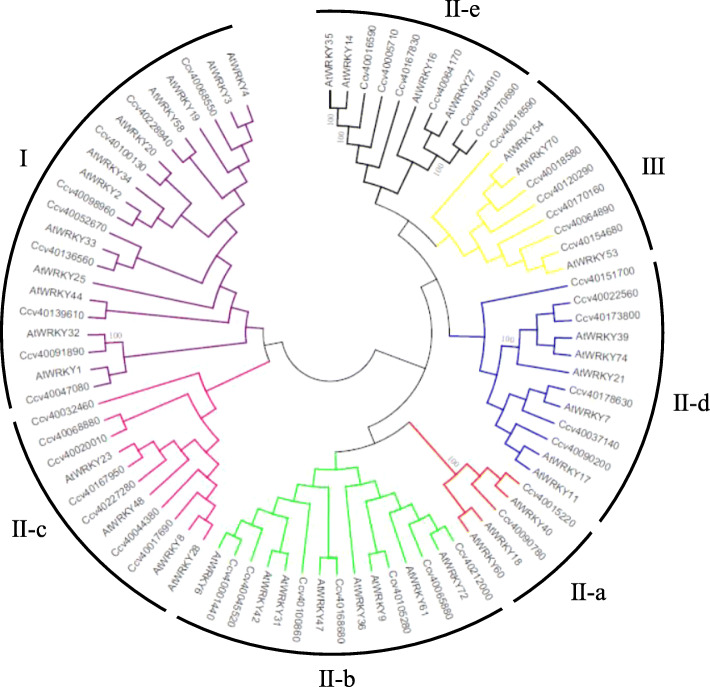
Unrooted phylogenetic tree representing relationships among WRKY domains of jute and *Arabidopsis*. The unrooted tree was constructed using the software of MEGA7.0 with the neighbor-joining method. Bootstrap values were calculated for 1000 replicates. The black arcs indicate different groups (or subgroups) of WRKY domains. “AtWRKYs” are the WRKY proteins from *Arabidopsis*

By comparison of the WRKY TFs of *A. thaliana* with *CcWRKYs*, analysis of phylogenetic relationship showed that these *CcWRKYs* could be divided into three groups: I, II and III. And Group II can be divided into II-a, II-b, II-c, II-d and II-e subgroups (Fig. 3). The phylogenetic tree analysis confirmed the results of the conservative domain analysis of *CcWRKYs* (Fig. 2**,** Table 1).

### Gene structure analysis of *CcWRKYs*

**Fig. 4:**
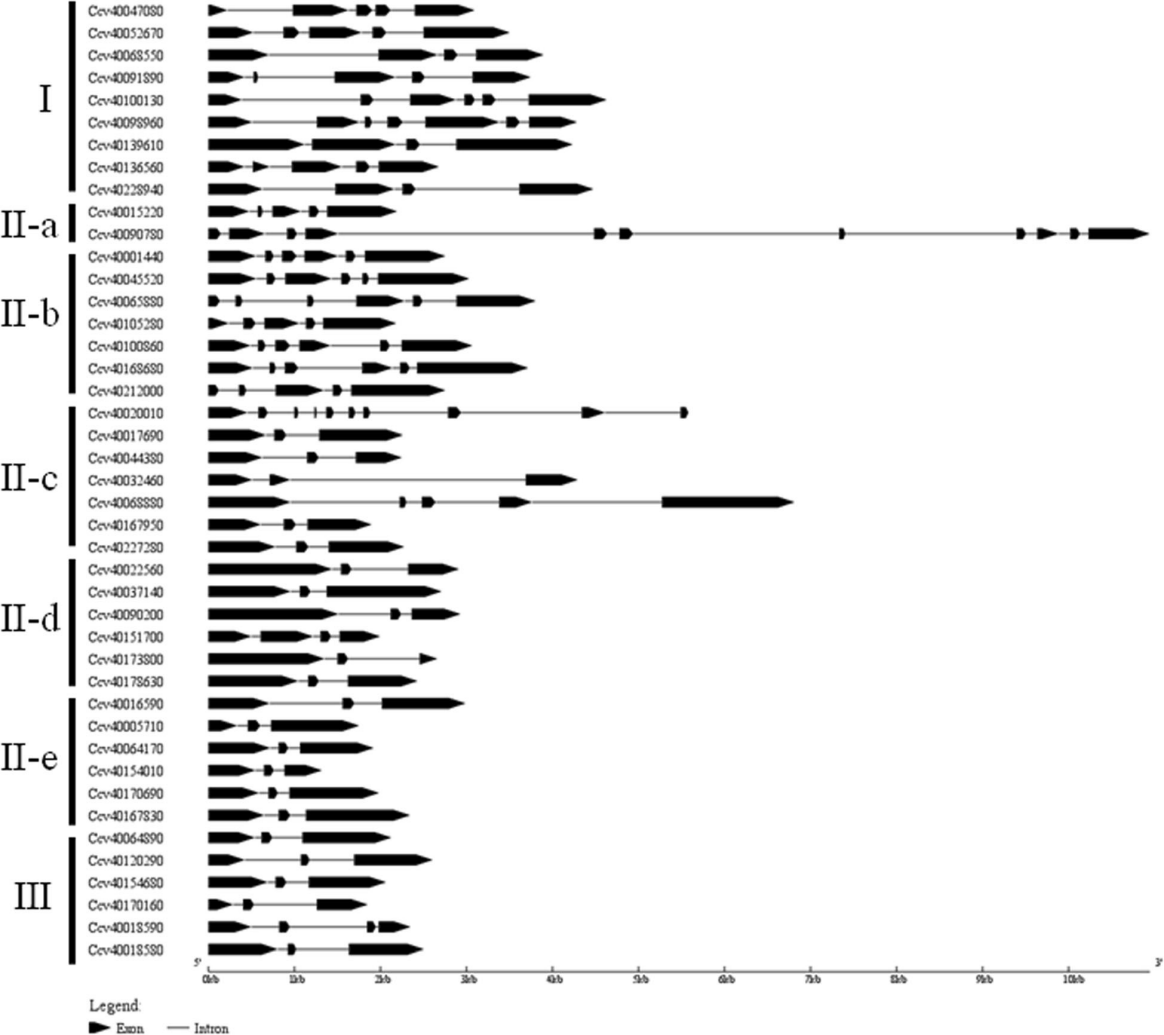
The gene structures of *CcWRKYs*. It shows the exon (black box)-intron (black lines) organization of the same genes. Exon-intron structure analyses of *CcWRKY* genes were performed by the online tool GSDS

The number of exons and introns of *CcWRKYs* were analyzed, as shown in Fig. 4. The number of exons varied from 3 to 11. 21 WRKYs (48.84%) contained 3 exons, 5 WRKYs (11.63%) contained 4 exons, 8 WRKYs (18.60%) contained 5 exons, and 6 WRKYs (13.95%) contained 6 exons. From the different groups, the exon numbers of group II c + d + e and group III were relatively conservative, while the exon numbers of group I and group II a + b + c’s were significantly different and varied greatly. Most of *CcWRKYs* in group II c + d + e and group III contain 3 exons except Ccv40151700 (4 exons) and Ccv40018590 (4 exons).

The tertiary structure of WRKYs is further coiled and folded based on the secondary structure. The tertiary structures of *CcWRKYs* were conducted by SWISS-MODEL. The majority of the 43 *CcWRKYs* have the similar three-dimensional structure. One representative homology modeling from *CcWRKYs* was shown in Additional file 8 (Fig. S1), consisting of several beta folding. This tertiary structure was similar to that of *Arabidopsis thaliana* [33], which confirmed that the *CcWRKYs* are highly conserved in the tertiary structure.

### Expression analysis of *CcWRKYs* in different stem growth stages

Tissue-specific expression of genes is often considered as markers of specific gene functions in this tissue. Since WRKY genes are related to the bast fiber development of plants [34, 35], the expression of *CcWRKYs* in different stages of stem growth was analyzed. R language was used to draw the heatmap of the expression patterns of *CcWRKYs* in different stem growth stages (Additional file 9: Fig. S2) based on the RNA-seq data. The difference of gene expression is generally represented by different colors, that mean the red color represents high expression and the blue one represents low expression. All the *CcWRKYs* were expressed in different stages of stem growth, while the expression of *CcWRKYs* differ in various stem growth stages. It indicates that there were no pseudogenes in 43 *CcWRKYs*. From the expression pattern of the heatmap, 43 *CcWRKYs* were divided into two categories (Additional file 9: Fig. S2). The expression of 13 of 43 *CcWRKYs* was low in the different tissues of jute, while the others were high. Totally, 10 *CcWRKYs* were highly expressed in Leaf-60d, 3 *CcWRKYs* were highly expressed in Hypocotyl-10d, 2 *CcWRKYs* were highly expressed in Stem stick-60d, 2 *CcWRKYs* were highly expressed in Stem bark-60d, 14 *CcWRKYs* were highly expressed in Root-60d, and 12 *CcWRKYs* were highly expressed in Stem bark-120d. It could be seen that these *CcWRKYs* were mainly expressed in the stem barks of jute. With the continuous stem growth stages of jute, the bast fiber of jute will gradually accumulate in the stem bark. Therefore, it is believed reasonably that the WRKY genes may be involved in bast fiber development in jute. For example, Ccv40032460 was highly expressed in Hypocotyl-10d, lowly expressed in Stem bark-60d, and no expression in Stem bark-120d. It suggests that this gene might play a negative regulatory role in jute fiber accumulation.

### Expression pattern of GA_3_ stress-responsive *CcWRKY* genes in the vegetative growth period

After being treated with MS culture media of 0.1 mg·L^− 1^ exogenous GA_3_, the hypocotyl length of “Aidianyehuangma” showed significant difference compared with “Aidianyehuangma” without being treated with exogenous hormone, and reached that of an elite cultivar “Huangma 179” (Additional file 10: Fig. S3). “Aidianyehuangma” is a typical GA_3_ sensitive dwarf germplasm, as shown in our previous study [36]. Since bioactive gibberellins (GAs) play the key roles in stem development, the two genotypes of “Aidianyehuangma” and “Huangma 179”, were used to characterize the expression pattern of these *CcWRKYs* in bast fibers in response to GA_3_ stress in the vegetative growth period (60 DAS).

**Fig. 5:**
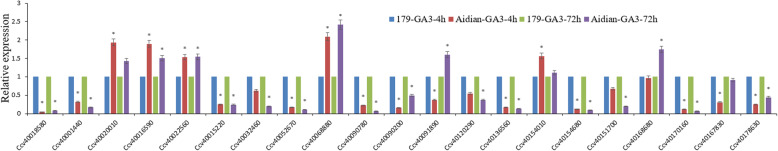
Expression analyses of *CcWRKYs* in a typical GA_3_ sensitive dwarf germplasm “Aidianyehuangma” in response to GA_3_ stress. The transcript levels were determined in stem barks from the data of RNA-seq in response to GA_3_ stress. Error bars indicate standard deviation of the means. Data points marked with asterisk (FDR < 0.05 & |log2(fold change) | > 1) show significant differences between the elite cultivar “Huangma 179” and GA_3_ sensitive dwarf germplasm “Aidianyehuangma” in response to GA_3_ stress. 179: the elite cultivar “Huangma 179”, Aidian: a GA_3_ sensitive dwarf germplasm “Aidianyehuangma”, GA3-4 h: After 4 h of the GA_3_ stress treatment, GA3-72 h: After 72 h of the GA_3_ stress treatment

The stem barks were treated with GA_3_ stress for “Huangma 179” and “Aidianyehuangma” on 60 DAS. Then, the stem barks were sampled in two different time points of 4 and 72 h later after spraying GA_3_, respectively. The stem barks without GA_3_ treatment were used as control. The RNA-seq data of these samples were analyzed, and then drew the corresponding histogram using FPKM (Additional file 11: Fig. S4, Additional file 12: Fig. S5). The above x-axis columns indicated that the gene expressions were up-regulated, while the below x-axis ones showed that the gene expressions were down-regulated. The expression of most *CcWRKYs* was regulated significantly under GA_3_ stress, especially for the down regulated genes. By comparison of the expression of *CcWRKYs* under different time points (4 and 72 h later) after spraying GA_3_, the expression of 33 of 43 *CcWRKYs* in the stem barks was regulated in the same trend in both of “Huangma 179” and “Aidianyehuangma”. Among them, it found that 21 *CcWRKYs* were regulated significantly under the GA_3_ stress in the stem barks of “Aidianyehuangma” compared with “Huangma 179” (Fig. 5**and** Additional file 13**: Table S8**), except Ccv40020010, Ccv40015220, Ccv40068880, Ccv40154010 and Ccv40090780. Among these 21 *CcWRKYs*, the transcript levels of 14 *CcWRKYs* were down-regulated and 7 *CcWRKYs* were up-regulated in the stem barks under the GA_3_ stress. To verify the accuracy of the gene expression, 9 *CcWRKYs* were randomly selected for RT-qPCR analysis (Additional file 14: Fig. S6). RT-qPCR assays of the expression patterns of the 9 *CcWRKYs* were similar to the results of FPKM.

### Evaluation of GA_3_ stress-responsive CcWRKY genes involved in cell wall formation

To evaluate GA_3_ stress-responsive CcWRKY genes involved in cell wall formation, some secondary cell wall biosynthesis genes, including *CesA1* (CesA, Cellulose synthase), *CesA4*, *CesA7*, *CesA8*, *CCoAOMT* (Caffeioyl coenzyme A methyltransferase), and *4CL* (4-Coumarate: Coenzyme A Ligase) were selected as marker genes. And a few gibberellin biosynthesis genes, i.e., Ent-copalyl diphosphate synthase, Ent-kaurene oxidase, Ent-kaurene synthase, Ent-kaurenoic acid oxidase, GA 20-oxidase, Gibberellin 2,3-hydroxylase and Gibberellin C13 oxidase, were also screened.

**Fig. 6:**
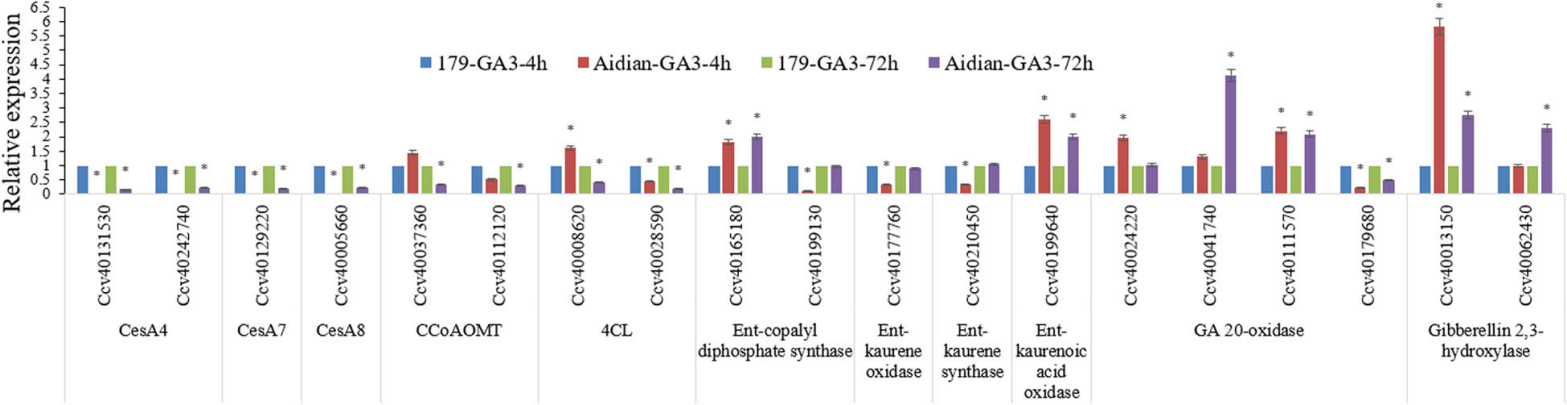
Expression analyses of several secondary cell wall biosynthesis genes in a typical GA_3_ sensitive dwarf germplasm “Aidianyehuangma” in response to GA_3_ stress. The transcript levels were determined in stem barks from the data of RNA-seq in response to GA_3_ stress. Error bars indicate standard deviation of the means. Data points marked with asterisk (FDR < 0.05 & |log2(fold change) | > 1) show significant differences between the elite cultivar “Huangma 179” and GA_3_ sensitive dwarf germplasm “Aidianyehuangma” in response to GA_3_ stress. 179: the elite cultivar “Huangma 179”, Aidian: a GA_3_ sensitive dwarf germplasm “Aidianyehuangma”, GA3-4 h: After 4 h of the GA_3_ stress treatment, GA3-72 h: After 72 h of the GA_3_ stress treatment

The variations of expression of most of these gibberellin biosynthesis genes in “Aidianyehuangma”(test: a typical GA_3_ sensitive dwarf germplasm) were more significant than those in “Huangma 179” (control: an elite cultivar) (Fig. 6**and** Additional file 15**: Table S9**), indicating GA_3_ play a certain role in stem development according to the gene expression regulation of these gibberellin biosynthesis genes. Notably, expression of most of these secondary cell wall biosynthesis genes in “Aidianyehuangma” were down-regulated significantly than those in “Huangma 179” (Fig. 6). The lignin synthase genes (*CCoAOMT* and *4CL*) and cellulose synthase genes (*CesA4*, *CesA7* and *CesA8*) showed down-regulation of their expression in “Aidianyehuangma” compared with “Huangma 179”. Combined with expression pattern of 21 GA_3_ stress-responsive *CcWRKYs* (Fig. 5), it gives a hint that the 7 up-regulated *CcWRKYs* may play a positive role in response to GA_3_ stress while the 14 down-regulated *CcWRKYs* might play a negative role in the development of stem barks of “Aidianyehuangma” under the GA_3_ stress.

To confirm this hint, *cis*-element analysis of promoters of these 21 *CcWRKYs* were analyzed using PlantCARE (Table 2). The promoters of these 21 *CcWRKYs* had 1 to 4 *cis*-elements involved in gibberellin-responsiveness, such as GARE-motif, P-box and TATC-box. These 21 *CcWRKYs* were responded to the stress of GA_3_ and could increase the fiber related traits including plant height of the “Aidianyehuangma”, suggesting that they might be involved in the development of bast fiber in jute in response to GA_3_ stress.

**Table 2:** Prediction of gibberellin-responsive cis-elements in the upstream regions of jute WRKY genes

GeneBank ID	Cis-elements	Sequence (5′-3′)	Function	No.^*^
Ccv40018580	TATC-box	TATCCCA	cis-acting element involved in gibberellin-responsiveness	1
Ccv40018580	GARE-motif	TCTGTTG	gibberellin-responsive element	1
Ccv40001440	P-box	CCTTTTG	gibberellin-responsive element	3
Ccv40001440	GARE-motif	TCTGTTG	gibberellin-responsive element	2
Ccv40016590	P-box	CCTTTTG	gibberellin-responsive element	3
Ccv40015220	P-box	CCTTTTG	gibberellin-responsive element	1
Ccv40052670	TATC-box	TATCCCA	cis-acting element involved in gibberellin-responsiveness	1
Ccv40068880	P-box	CCTTTTG	gibberellin-responsive element	2
Ccv40068880	TATC-box	TATCCCA	cis-acting element involved in gibberellin-responsiveness	2
Ccv40068880	GARE-motif	TCTGTTG	gibberellin-responsive element	2
Ccv40090780	GARE-motif	TCTGTTG	gibberellin-responsive element	2
Ccv40090200	P-box	CCTTTTG	gibberellin-responsive element	1
Ccv40091890	P-box	CCTTTTG	gibberellin-responsive element	2
Ccv40170160	TATC-box	TATCCCA	cis-acting element involved in gibberellin-responsiveness	1
Ccv40170160	P-box	CCTTTTG	gibberellin-responsive element	1
Ccv40178630	TATC-box	TATCCCA	cis-acting element involved in gibberellin-responsiveness	1
Ccv40178630	P-box	CCTTTTG	gibberellin-responsive element	1
Ccv40178630	GARE-motif	TCTGTTG	gibberellin-responsive element	1
Ccv40020010	P-box	CCTTTTG	gibberellin-responsive element	2
Ccv40154680	TATC-box	TATCCCA	cis-acting element involved in gibberellin-responsiveness	1
Ccv40154680	GARE-motif	TCTGTTG	gibberellin-responsive element	1
Ccv40154680	P-box	CCTTTTG	gibberellin-responsive element	1
Ccv40032460	P-box	CCTTTTG	gibberellin-responsive element	2
Ccv40120290	P-box	CCTTTTG	gibberellin-responsive element	1
Ccv40120290	GARE-motif	TCTGTTG	gibberellin-responsive element	2
Ccv40154010	P-box	CCTTTTG	gibberellin-responsive element	4
Ccv40154010	TATC-box	TATCCCA	cis-acting element involved in gibberellin-responsiveness	1
Ccv40151700	GARE-motif	TCTGTTG	gibberellin-responsive element	1
Ccv40151700	P-box	CCTTTTG	gibberellin-responsive element	3
Ccv40151700	TATC-box	TATCCCA	cis-acting element involved in gibberellin-responsiveness	2
Ccv40168680	P-box	CCTTTTG	gibberellin-responsive element	4
Ccv40168680	TATC-box	TATCCCA	cis-acting element involved in gibberellin-responsiveness	1
Ccv40168680	GARE-motif	TCTGTTG	gibberellin-responsive element	1
Ccv40167830	GARE-motif	TCTGTTG	gibberellin-responsive element	1
Ccv40167830	P-box	CCTTTTG	gibberellin-responsive element	2
Ccv40167830	TATC-box	TATCCCA	cis-acting element involved in gibberellin-responsiveness	1

Note: ^*^Numbers of the cis-elements

## Discussion

### CcWRKY transcription factors in jute

WRKY transcription factors are one of the largest families of transcriptional regulators in plants. They play important roles in plant development, biotic and abiotic stresses [1, 37–39]. The present study identified a total of 43 WRKY genes in the whole genome of jute. The number of WRKY genes in jute was consistent with that in sugar beet (40) [40], canola (43) [41] and castor (47) [42]. However, it was inconsistent to that in other plants’ genomes such as *Arabidopsis thaliana* (74), cotton (116) [16], soybean (188) [43], poplar (104) [15], tomato (81) [44], cabbage (145) [45], whose members were more than those of jute. By comparing with different species, the numbers of WRKY genes in different species are not proportional to their genome size. Recently, researchers have suggested that gene duplication, segmental duplication and whole genome duplication play important roles in the mass production of gene families [43]. Unfortunately, due to the insufficient data on jute researches, particularly genome data. The only published draft jute genome was not enough to provides satisfactory solutions to many problems. We suspected that jute genome WRKY genes were less than that in other species, perhaps because they did not experience whole genome replication as other species do. However, with the improvement of genome sequencing and assembly, we believed that the new WRKY members also existed in the genome of jute.

In general, the locations of introns and exons in the genome may provide important evidences for their evolutionary relationships. In this study, it was comprehensively to analyze the distributions and lengths of exons and introns of *CcWRKYs*. By analyzing the gene structures of *CcWRKYs*, it was found that its members consisted of 3 to 11 exons, and nearly half of them were 3 exons. Moreover, the members of the WRKY gene family in jute have similar three-dimensional structures, which were formed by several beta folds. It is similar to the 3D structure of the domains of *Arabidopsis* WRKY protein in the database [33]. These results provided valuable information for the evolutions of the WRKY gene family in various species.

### GA_3_ stress-responsive CcWRKY genes involved in cell wall formation

Group III genes, one among the WRKY gene family have played a vital role in plant evolution and adaptions, hence it is regarded as the most advanced in respect of evolution [46]. However, according to the expression analysis of *CcWRKYs* in different tissues, it could be found that most of *CcWRKYs* in group III (Ccv40018580, Ccv40120290, Ccv40170160 and Ccv40154680) were highly expressed during the fiber development except two genes (Ccv40018590 and Ccv40064890). Theses results in jute were consistent to that in cotton [47]. It is suggested that *CcWRKYs* in group III in jute may have certain functional differentiation in fiber crops, which may have certain influence on the fiber development of crops. In recent years, the study of Wang et al. [48] has shown that *Arabidopsis* WRKY transcription factors were involved in the formation of secondary cell wall, which could significantly increase plant biomass, such as *AtWRKY12*. In this study, most of the CcWRKY genes expressed differently in the stem barks and hypocotyls. This interesting phenomenon leaded us to believe that there was a certain connection between WRKY transcription factors and bast fiber development.

Phytohormones are known to play a significant role in plant cellulose biosynthesis. GA is a key hormone for the growth and development of plant in its entire life cycle. Gibberellin has a great effect on the initiation, differentiation and development of cotton fiber, which can induce more fiber initial cells and stimulate fiber elongation. Therefore, the gene expression patterns of 43 *CcWRKYs* under GA_3_ stress in this study were systematically analyzed. Because of the great influences of GA_3_ on plant heights, a typical GA_3_ sensitive dwarf germplasm (“Aidianyehuangma”) was selected as the target of GA_3_ stress treatment. Expression pattern showed that the expression of 21 of 43 *CcWRKYs* was significantly regulated in “Aidianyehuangma”. It indicated that these *CcWRKYs* were likely to be involved in bast fiber development under GA_3_ stress. At present, it has been shown that gibberellin-mediated signaling cascade regulates cellulose synthesis [49]. Therefore, mutations in Gibberellin gene could change transcription of CesAs (cellulose synthase) genes. And GA could regulate secondary wall cellulose synthesis in land plants. Previous studies reported that WRKY transcription factors were involved in epidermal hair development, glucose and gibberellin signaling [50]. Therefore, it will be very interesting to further study about the relationships between CcWRKY genes, GA_3_ stress and fiber development.

In addition, due to the economic importance of jute, it will be very important to study the transcriptional regulation of WRKYs for jute improvement. These CcWRKY genes have obvious effects on fiber development and response to GA_3_ stress. At present, the research of transcription factors in *Arabidopsis thaliana* was only a part of them, and it couldn’t fully reveal the functions of each transcription factor. And the same transcription factors have different functions in different species because of the different evolutionary directions. Overall, this study provided a theoretical basis for the subsequent study of the CcWRKY genes and their effects on fiber development by identification and bioinformatics analysis of the CcWRKY genes.

## Conclusion

De novo assembly of transcriptome of 12 sequencing libraries was conducted to obtain and characterize the expression pattern of GA_3_ stress-responsive WRKY transcription factors in jute. Totally, 43 *CcWRKYs* in jute were identified. The numbers of exons of these CcWRKY genes varied widely, indicating they may have a functional diversity. Expression profiles of these *CcWRKYs* showed a variety of expression patterns at different stages of stem development. 21 of 43 *CcWRKYs* was regulated significantly with secondary cell wall biosynthesis genes in response to GA_3_ stress. This suggested that these *CcWRKYs* might be involved in the growth and development of bast fiber in jute.

## Supplementary information

**Additional file 1: Table S1.** RNA samples for jute (*C. capsularis*) in this study.

**Additional file 2: Table S2.** Summary statistics of the transcriptome sequence of jute.

**Additional file 3: Table S3.** Transcripts and Unigenes length interval and their distributions.

**Additional file 4: Table S4.** Splicing length distribution.

**Additional file 5: Table S5.** Unigenes annotation in the public databases.

**Additional file 6: Table S6.** Annotated unigenes in the Nr databases.

**Additional file 7: Table S7.** Variation of the heptapeptide WRKYGQK and zinc-finger structure in WRKY domains of *CcWRKYs*.

**Additional file 8: Figure S1.** Protein 3D structure prediction of WRKY family in jute.

**Additional file 9: Figure S2.** Expression profiles of *CcWRKYs* in different tissues and developmental stages. FPKM values of *CcWRKY* genes were transformed by log2 and the heatmap was constructed by R language.

**Additional file 10: Figure S3.** Comparison of average hypocotyl length of the seedling of “Huangma 179” and “Aidianyehuangma” treated with GA_3_. The standard deviation is plotted using vertical lines (*P < 0.05, Student’s *t*-test).

**Additional file 11: Figure S4.** Transcript abundances of *CcWRKYs* after GA_3_ stress. The relative expression levels of *CcWRKY* genes in each treated time point of each variety were compared with that in each time point at normal conditions. The standard deviation is plotted using vertical lines. The differentially expressed genes among the treated samples were estimated by referring to the standard of FDR < 0.05 and |log2(fold change) | > 1. 179: elite cultivar “Huangma 179”, Aidian: GA_3_ sensitive dwarf germplasm “Aidianyehuangma”, GA3-4 h: After 4 h of the GA_3_ stress treatment, GA3-72 h: After 72 h of the GA_3_ stress treatment.

**Additional file 12: Figure S5.** Transcript abundances of secondary wall biosynthesis genes after GA_3_ stress. CesA: Cellulose synthase; CCoAOMT: Caffeioyl coenzyme A methyltransferase; 4CL: 4-Coumarate: Coenzyme A Ligase. The relative expression levels of secondary wall biosynthesis genes in each treated time point of each variety were compared with that in each time point at normal conditions. The standard deviation is plotted using vertical lines. The differentially expressed genes among the treated samples were estimated by referring to the standard of FDR < 0.05 and |log2(fold change) | > 1. 179: elite cultivar “Huangma 179”, Aidian: GA_3_ sensitive dwarf germplasm “Aidianyehuangma”, GA3-4 h: After 4 h of the GA_3_ stress treatment, GA3-72 h: After 72 h of the GA_3_ stress treatment.

**Additional file 13: Table S8.** Expression levels of *CcWRKYs* from the elite cultivar “Huangma 179” and GA_3_ sensitive dwarf germplasm “Aidianyehuangma” in response to GA_3_ stress.

**Additional file 14: Figure S6.** RT-qPCR assays of the expression patterns of 9 *CcWRKYs* in response to GA_3_ treatments. Data are means ± SD calculated from three biological replicates. 179: elite cultivar “Huangma 179”, Aidian: GA_3_ sensitive dwarf germplasm “Aidianyehuangma”, GA3-4 h: After 4 h of the GA_3_ stress treatment, GA3-72 h: After 72 h of the GA_3_ stress treatment.

**Additional file 15: Table S9.** Expression levels of several secondary cell wall biosynthesis genes from the elite cultivar “Huangma 179” and GA_3_ sensitive dwarf germplasm “Aidianyehuangma” in response to GA_3_ stress.

## Data Availability

Raw Illumina sequence data were deposited in the National Center for Biotechnology Information (NCBI) and be accessed in the sequence read archive (SRA) database (https://www.ncbi.nlm.nih.gov/sra). The accession number is PRJNA555734 (https://www.ncbi.nlm.nih.gov/bioproject/PRJNA555734). All data generated or analysed during this study are included in this published article and its supplementary information files.

## Abbreviations

GA_3_: Gibberellin
CcWRKY: Jute (*Corchorus capsularis*) WRKY
AtWRKY: *Arabidopsis thaliana* WRKY;
CesA: Cellulose synthase
CCoAOMT: Caffeioyl coenzyme A methyltransferase
4CL: 4-Coumarate: Coenzyme A Ligase
TF: Transcription factor
FPKM: Fragments Per Kilobase Per Million Mapped Reads
