# Supplementary figures and images for: De novo assembly of transcriptome and genome-wide identification reveal GA3 stress-responsive WRKY transcription factors involved in fiber formation in jute (Corchorus capsularis)
Source: BMC Plant Biol. 2020 Sep 1;20:403. doi: 10.1186/s12870-020-02617-8 (PMC7460746; doi:10.1186/s12870-020-02617-8)

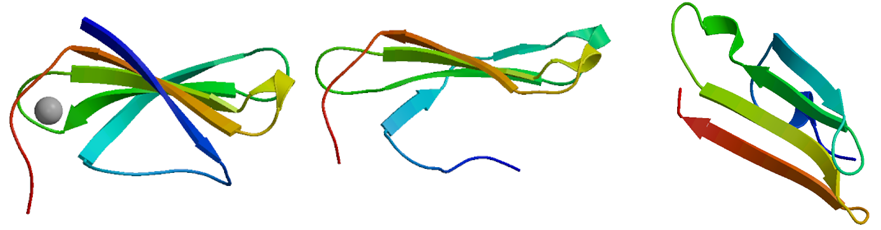

Supplement: Supplementary file 8 — Additional file 8: Figure S1. Protein 3D structure prediction of WRKY family in jute. [file 12870_2020_2617_MOESM8_ESM.png]

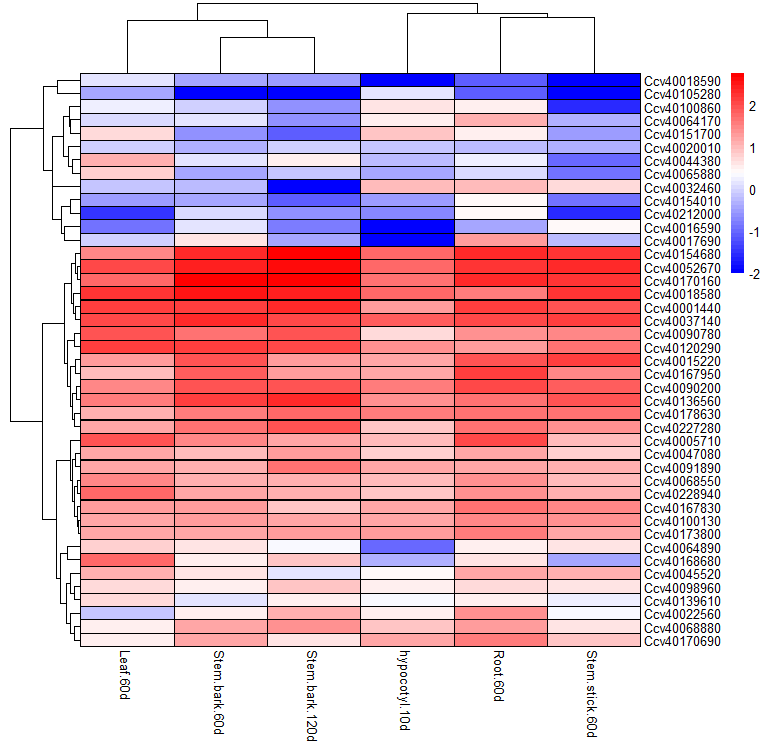

Supplement: Supplementary file 9 — Additional file 9: Figure S2. Expression profiles of CcWRKYs in different tissues and developmental stages. FPKM values of CcWRKY genes were transformed by log2 and the heatmap was constructed by R language. [file 12870_2020_2617_MOESM9_ESM.png]

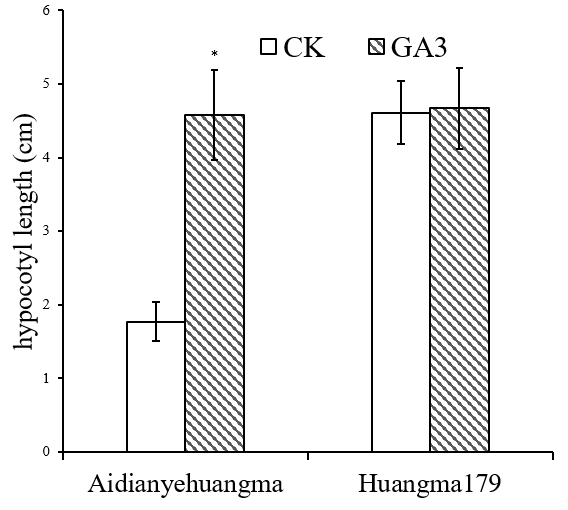

Supplement: Supplementary file 10 — Additional file 10: Figure S3. Comparison of average hypocotyl length of the seedling of “Huangma 179” and “Aidianyehuangma” treated with GA3. The standard deviation is plotted using vertical lines (*P < 0.05, Student’s t-test). [file 12870_2020_2617_MOESM10_ESM.png]

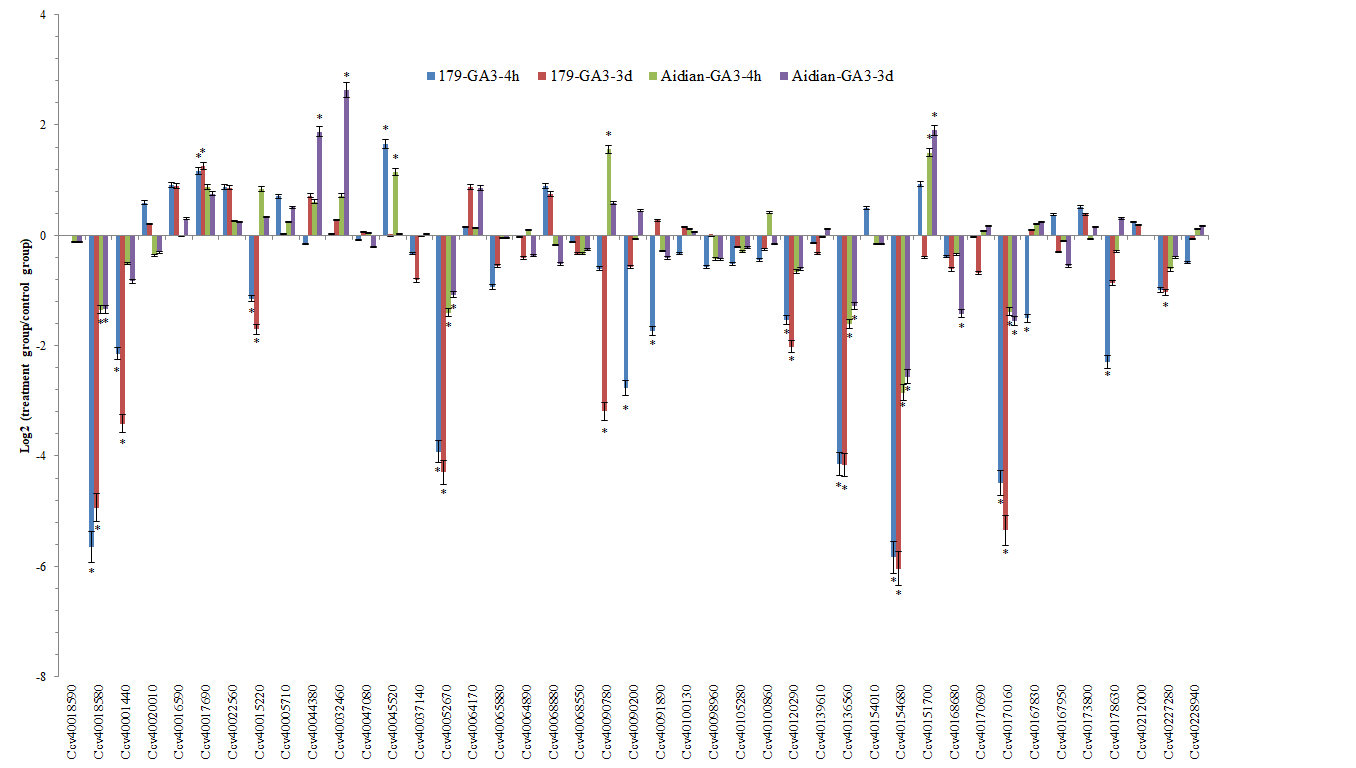

Supplement: Supplementary file 11 — Additional file 11: Figure S4. Transcript abundances of CcWRKYs after GA3 stress. The relative expression levels of CcWRKY genes in each treated time point of each variety were compared with that in each time point at normal conditions. The standard deviation is plotted using vertical lines. The differentially expressed genes among the treated samples were estimated by referring to the standard of FDR < 0.05 and |log2(fold change) | > 1. 179: elite cultivar “Huangma 179”, Aidian: GA3 sensitive dwarf germplasm “Aidianyehuangma”, GA3-4 h: After 4 h of the GA3 stress treatment, GA3-72 h: After 72 h of the GA3 stress treatment. [file 12870_2020_2617_MOESM11_ESM.png]

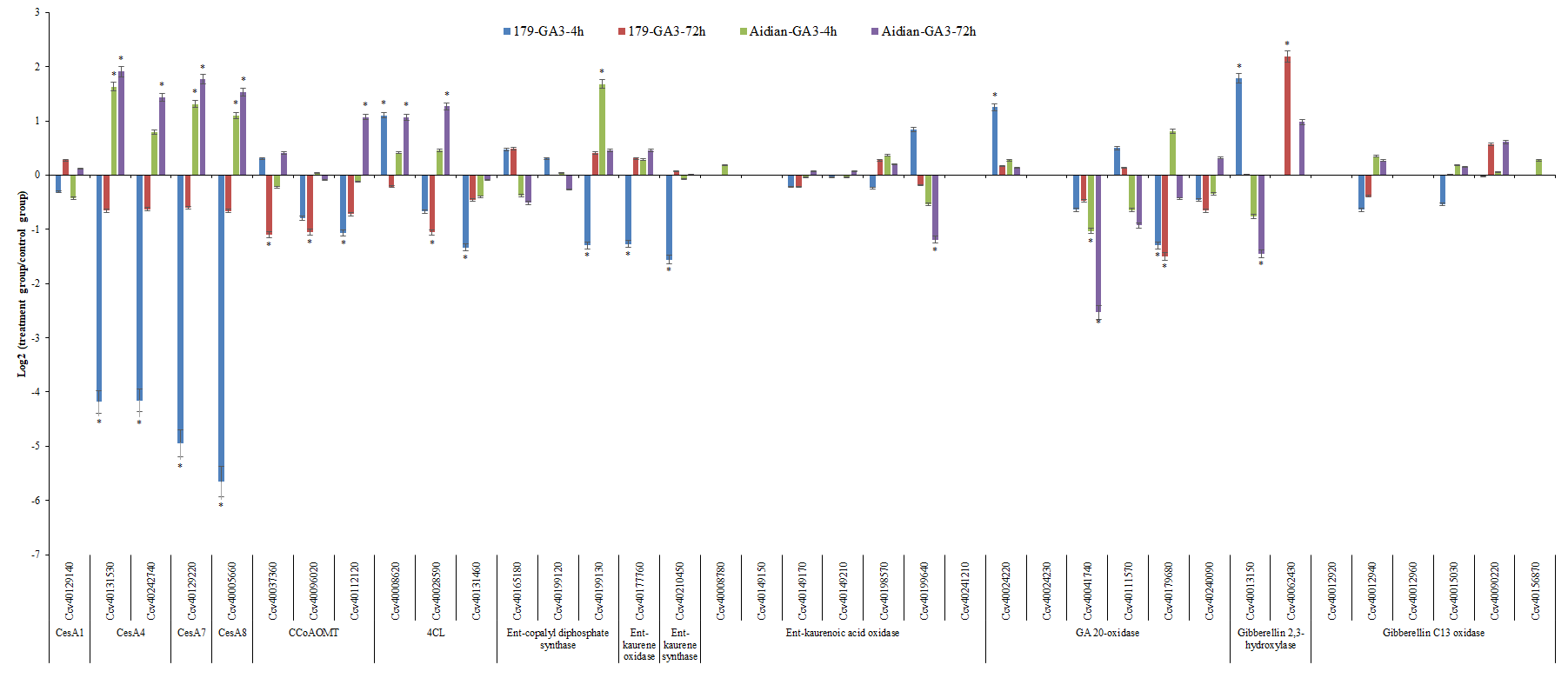

Supplement: Supplementary file 12 — Additional file 12: Figure S5. Transcript abundances of secondary wall biosynthesis genes after GA3 stress. CesA: Cellulose synthase; CCoAOMT: Caffeioyl coenzyme A methyltransferase; 4CL: 4-Coumarate: Coenzyme A Ligase. The relative expression levels of secondary wall biosynthesis genes in each treated time point of each variety were compared with that in each time point at normal conditions. The standard deviation is plotted using vertical lines. The differentially expressed genes among the treated samples were estimated by referring to the standard of FDR < 0.05 and |log2(fold change) | > 1. 179: elite cultivar “Huangma 179”, Aidian: GA3 sensitive dwarf germplasm “Aidianyehuangma”, GA3-4 h: After 4 h of the GA3 stress treatment, GA3-72 h: After 72 h of the GA3 stress treatment. [file 12870_2020_2617_MOESM12_ESM.png]

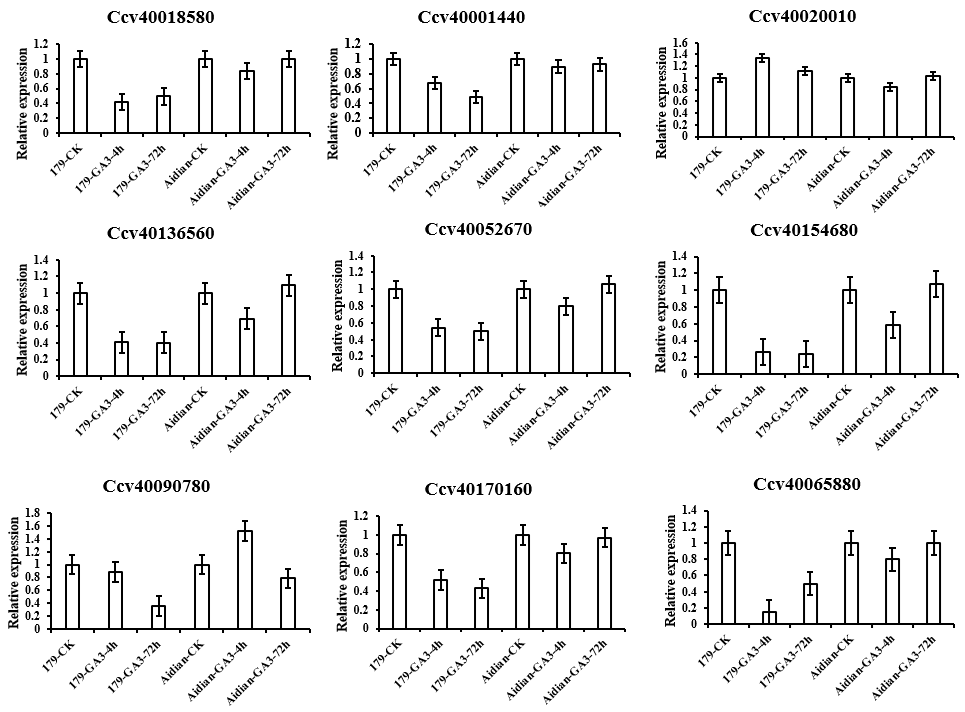

Supplement: Supplementary file 14 — Additional file 14: Figure S6. RT-qPCR assays of the expression patterns of 9 CcWRKYs in response to GA3 treatments. Data are means ± SD calculated from three biological replicates. 179: elite cultivar “Huangma 179”, Aidian: GA3 sensitive dwarf germplasm “Aidianyehuangma”, GA3-4 h: After 4 h of the GA3 stress treatment, GA3-72 h: After 72 h of the GA3 stress treatment. [file 12870_2020_2617_MOESM14_ESM.png]
